# Development and Preclinical Evaluation of a Copper-64-Labeled Antibody Targeting Glycine-Alanine Dipeptides for PET Imaging of C9orf72-Associated Amyotrophic Lateral Sclerosis/Frontotemporal Dementia

**DOI:** 10.1021/acsptsci.4c00037

**Published:** 2024-04-25

**Authors:** Monireh Shojaei, Qihui Zhou, Giovanna Palumbo, Rebecca Schaefer, Janne Kaskinoro, Pirjo Vehmaan-Kreula, Peter Bartenstein, Matthias Brendel, Dieter Edbauer, Simon Lindner

**Affiliations:** † Department of Nuclear Medicine, University Hospital, LMU Munich, 81377 Munich, Germany; ‡ German Center for Neurodegenerative Diseases (DZNE), 81377 Munich, Germany; § Orion Corporation Orion Pharma, 02200 Espoo, Finland; ∥ Munich Cluster for Systems Neurology (SyNergy), 81377 Munich, Germany

**Keywords:** Copper-64, mAb1A12, PET/CT, poly-GA, FTD/ALS

## Abstract

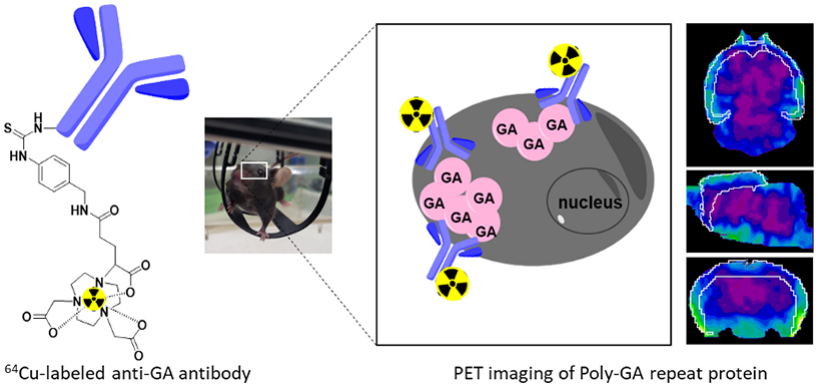

Aggregating poly(glycine-alanine) (poly-GA) is derived from the unconventional translation of the pathogenic intronic hexanucleotide repeat expansion in the *C9orf72* gene, which is the most common genetic cause of frontotemporal dementia (FTD) and amyotrophic lateral sclerosis (ALS). Poly-GA accumulates predominantly in neuronal cytoplasmic inclusions unique to *C9orf72* ALS/FTD patients. Poly-GA is, therefore, a promising target for PET/CT imaging of FTD/ALS to monitor disease progression and therapeutic interventions. A novel ^64^Cu-labeled anti-GA antibody (mAb1A12) targeting the poly-GA protein was developed and evaluated in a transgenic mouse model. It was obtained with high radiochemical purity (RCP), radiochemical yield (RCY), and specific activity, and showed high stability *in vitro* and *ex vivo* and specifically bound to poly-GA. The affinity of NODAGA-mAb1A12 for poly-GA was not affected by this modification. [^64^Cu]Cu-NODAGA-mAb1A12 was injected into transgenic mice expressing GFP-(GA)_175_ in excitatory neurons driven by Camk2a-Cre and in control littermates. PET/CT imaging was performed at 2, 20, and 40 h post-injection (p.i.) and revealed a higher accumulation in the cortex in transgenic mice than in wild-type mice, as reflected by higher standardized uptake value ratios (SUVR) using the cerebellum as the reference region. The organs were isolated for biodistribution and *ex vivo* autoradiography. Autoradiography revealed a higher cortex-to-cerebellum ratio in the transgenic mice than in the controls. Results from autoradiography were validated by immunohistochemistry and poly-GA immunoassays. Moreover, we confirmed antibody uptake in the CNS in a pharmacokinetic study of the perfused tissues. In summary, [^64^Cu]Cu-NODAGA-mAb1A12 demonstrated favorable *in vitro* characteristics and an increased relative binding in poly-GA transgenic mice compared to wild-type mice *in vivo*. Our results with this first-in-class radiotracer suggested that targeting poly-GA is a promising approach for PET/CT imaging in FTD/ALS.

Frontotemporal dementia (FTD) is a clinical syndrome^1^ caused by the degeneration of frontal and temporal lobes.^2^ Depending on the affected regions, the main symptoms are changes in behavior and personality or speech and language deficits.^1,3,4^ Amyotrophic lateral sclerosis (ALS) is a related neurodegenerative disorder,^5^ characterized by loss of cortical and spinal motor neurons, which leads to progressive paralysis and ultimately respiratory failure.^2,5−7^ Both neurodegenerative diseases show genetic, clinical, and pathological overlap, e.g., ALS patients develop mild FTD-like symptoms and vice versa.^2,6^ A (G_4_C_2_)_n_ hexanucleotide repeat expansion upstream of the *C9orf72* (chromosome 9 open reading frame 72) coding region^1^ is the most common known genetic cause of ALS and FTD in the western world and is found in 5–10% of all patients.^2,6,8,9^ Unconventional translation of bidirectional repeat transcripts^6,10−12^ results in five different dipeptide repeat proteins (DPR): poly-GA, poly-GP, poly-PA,^4,13^ poly-GR, and poly-PR.^14^ Selective expression of poly-GA, -GR, and -PR causes toxicity in various cell and animal models. The DPR proteins accumulate in neuronal inclusions, predominantly in the cytoplasm and less commonly in the nucleus.^14^ Poly-GA inclusions are by far the most abundant and the other DPR proteins co-aggregate less commonly.^14−16^ Poly-GA can be transmitted from cell to cell and is a key driver of disease development.^17−19^ Poly-GA protein expression could contribute to cytoplasmic mislocalization and accumulation of phosphorylated TDP-43 in both patients and transgenic mice.^7,15,16,20^ The anti-GA antibody 1A12^21^ binds specifically to poly-GA proteins and thereby decreases cell-to-cell transmission and aggregation of poly-GA and subsequent TDP-43 mislocalization in cell culture.^7,17^ Antibody therapy and active vaccination targeting poly-GA are promising therapeutic strategies.^15,22^ Interestingly, poly-GA inclusions are already present prior to disease onset^23^ and may contribute to the long prodromal disease phase with atrophy detectable 20 years prior to clinical onset, but correlate poorly with the degree of neurodegeneration in symptomatic cases.^3,21,24^

Based on these data, visualizing poly-GA aggregates in living patients could improve our understanding of *C9orf72* pathogenesis by allowing longitudinal studies from prodromal to end-stage diseases. Moreover, PET imaging of poly-GA pathology would be an attractive pharmacodynamic biomarker for future clinical trials. In this work, we developed a new radiotracer based on mouse monoclonal anti-GA antibody 1A12 for positron emission tomography. The tracer is able to visualize regional poly-GA pathology in a conditional mouse model expressing poly-GA in the neocortex and hippocampus.

## Results and Discussion

### Radiolabeling Did Not Impair the Affinity or Stability of mAb1A12

To enable ^64^Cu-labeling, we chemically conjugated the chelator *p*-NCS-benzyl-NODAGA to lysine residues of the mAb1A12 IgG2a antibody, resulting in the formation of a stable thiourea bond. The number of chelators per antibody was determined to be 1–3 using an arsenazo assay^25^ (Supporting Figure S1).

**Figure 1:**
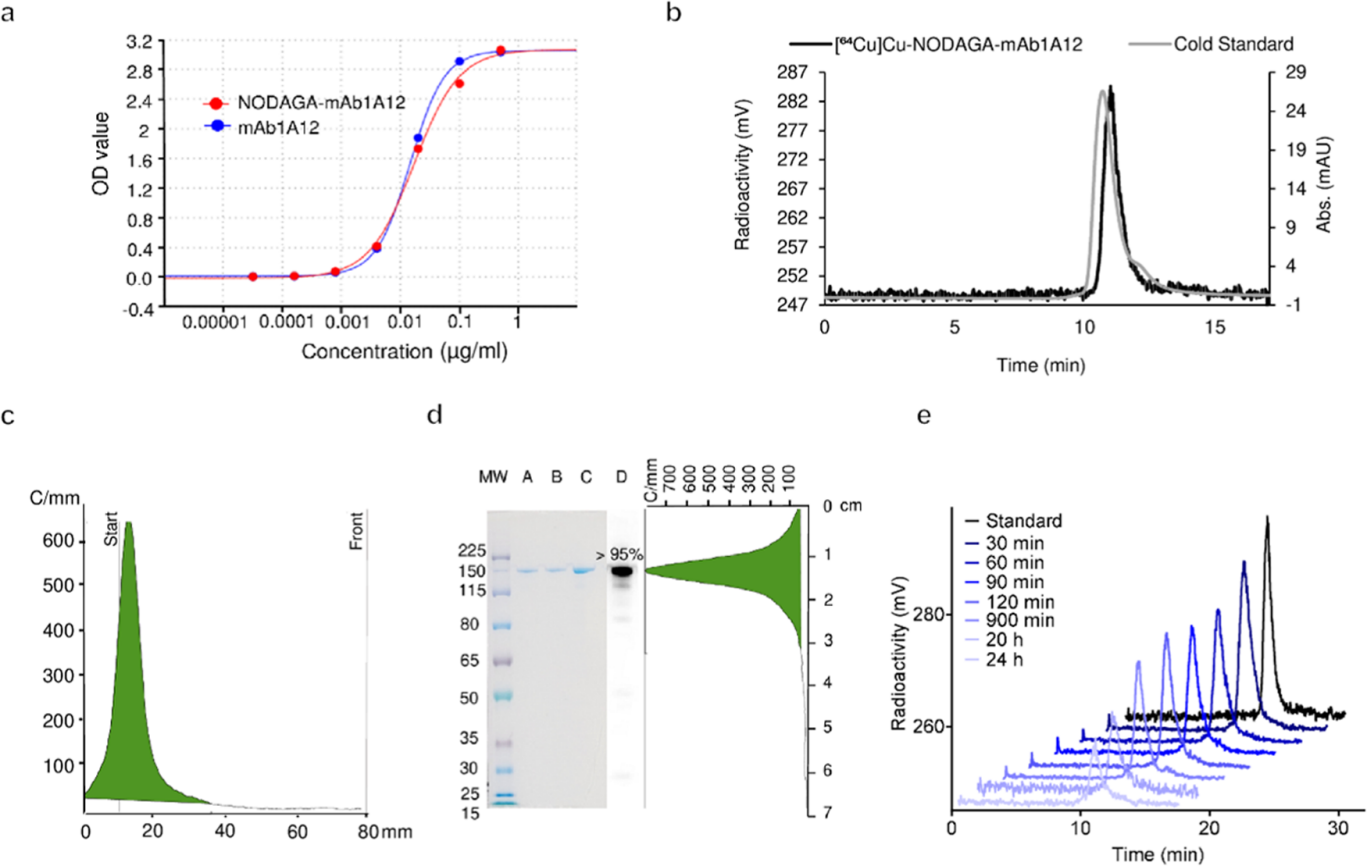
Characterization of NODAGA-mAb1A12 *in vitro*. (a) Determination of EC_50_ values for NODAGA-mAb1A12 (0.017 μg/mL) and mAb1A12 (0.015 μg/mL) by ELISA. (b) Representative HPLC chromatograms of cold standard (NODAGA-mAb1A12) in PBS, *R*_t_ = 10.8 min at 280 nm (UV channel) and [^64^Cu]Cu-NODAGA-mAb1A12, *R*_t_ = 11.0 min (radioactivity channel). (c) Radio-TLC of [^64^Cu]Cu-NODAGA-mAb1A12 on ITLC-SG chromatography paper, *R*_f_ (tracer) = 0.0–0.1; *R*_f_ (^64^Cu) = 0.9–1.0. (d) SDS-PAGE of [^64^Cu]Cu-NODAGA-mAb1A12 (A), NODAGA-mAb1A12 (B), and mAb1A12 (C) with autoradiography (D) and a radio-TLC scan of the SDS-PAGE gel. (e) Stability of [^64^Cu]Cu-NODAGA-mAb1A12 in murine plasma over 24 h, as measured by SEC-HPLC (radioactivity channel).

The functionality of the unmodified and modified antibodies (0.5 μg/mL) was verified by enzyme-linked immunosorbent assay (ELISA) using a dilution series of GST-(GA)_15_ antigen (Figure 1a). The EC_50_ values of NODAGA-mAb1A12 and mAb1A12 were comparable (0.017 vs 0.015 μg/mL). These results confirmed that antibody modification using NODAGA did not impair the binding affinity of mAb1A12.

Next, NODAGA-mAb1A12 was radiolabeled with [^64^Cu]CuCl_2_ (Figure 1b,1c). The [^64^Cu]Cu-NODAGA-mAb1A12 was obtained in high radiochemical purity (RCP) of 97.9 ± 1.9% (*n* = 5) and specific activity (A_s_) of 1.0 ± 0.3 MBq/μg (*n* = 8). No [^64^Cu]Cu-NODAGA complex or no free [^64^Cu]CuCl_2_ was observed in the final product (Supporting Figure S2). In addition, SDS-PAGE, subsequent autoradiography, and a radio-TLC scan of the SDS-PAGE gel confirmed the high purity of the modified and radiolabeled antibodies (Figure 1d). To assess tracer stability, [^64^Cu]Cu-NODAGA-mAb1A12 was incubated in mouse plasma, and stability was investigated by SEC-HPLC in a time series from 0.5 to 24 h. The tracer remained intact in the mouse plasma for at least 24 h (Figure 1e).

### [^64^Cu]Cu-NODAGA-mAb1A12 Detects Regional Poly-GA Pathology Using *In Vitro* Autoradiography

To confirm that the labeled antibody specifically targets cellular poly-GA inclusions, we used *in vitro* autoradiography combined with immunohistochemistry, taking advantage of a transgenic mouse model expressing high levels of GFP-(GA)_175_.

**Figure 2:**
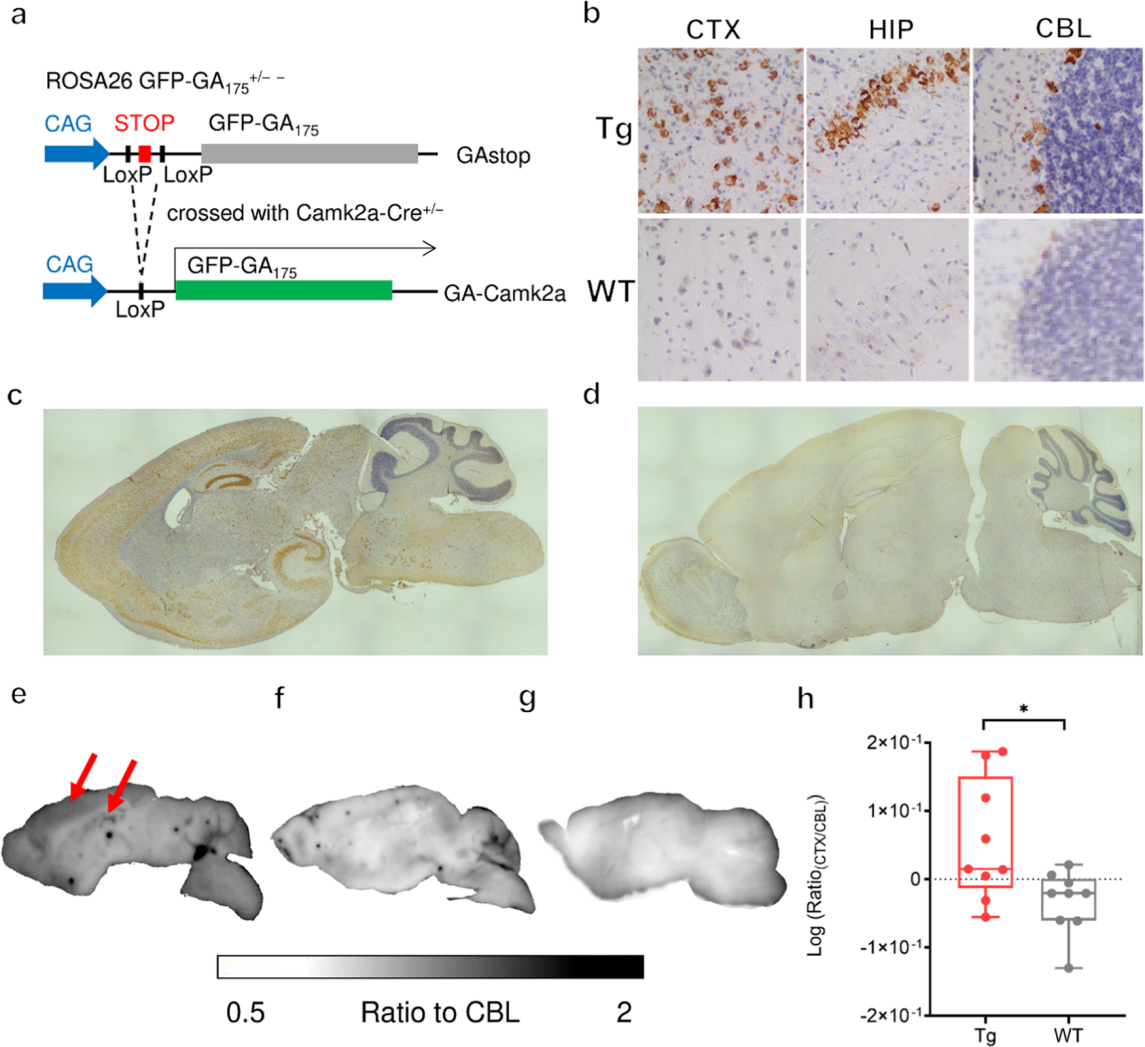
[^64^Cu]Cu-NODAGA-mAb1A12 specifically detects poly-GA pathology using autoradiography. (a) Schematic depiction of the generation of the GA^±^Camk2a^±^ Tg mouse model. (b) IHC analysis of the cortex, hippocampus, and cerebellum in Tg and WT mice with 20× magnification. IHC overviews of brain sections from (c) transgenic and (d) wild-type mice. *In vitro* autoradiography of sagittal brain sections of (e) GA^±^Camk2a^±^ Tg and (f) wild-type mice. Intense signals are observed in the neocortex and hippocampus of the Tg mice (arrows). (g) *In vitro* autoradiography of the brain sections of the transgenic mice treated with tracer and a 1000-fold excess of native antibody shows a complete block of the signal. (h) The brain sections of transgenic mice revealed a higher cortex-to-cerebellum ratio than wild-type mice (mean ± SD, unpaired *t*-test, *p* ≤ 0.05).

CNS-wide expression of the GFP-(GA)_175_ transgene using the Nestin-Cre driver^26^ results in weight loss and weakness requiring termination at 6–7 weeks of age. To allow experiments in adult mice and comparison of poly-GA-expressing and -nonexpressing regions within each animal, we used Camk2a-Cre to drive poly-GA expression specifically in excitatory neurons^27^ (Figure 2a). Immunohistochemistry (IHC) with mAb1A12 confirmed regional expression in the neocortex, striatum, and hippocampus with minimal staining in the cerebellum, consistent with the known expression pattern of Camk2a-Cre^26^ (Figure 2b–d). In addition, we quantified poly-GA levels in the brains of transgenic (Tg) mice using a poly-GA immunoassay (Supporting Figure S3 and Table S1). The highest poly-GA concentration was observed in the cortex and hippocampus, the lower levels in the midbrain and brainstem, and the lowest in the cerebellum. Control (WT) mice only showed negligible background staining.

We incubated sagittal brain sections from Tg and WT mice with [^64^Cu]Cu-NODAGA-mAb1A12. *In vitro* autoradiography showed elevated tracer accumulation in the cortex and hippocampus of GA^±^Camk2a^±^ Tg brain slices compared to wild-type (Figure 2e,2f), which is consistent with the results of IHC and poly-GA immunoassay. The cerebellum reveals unspecific uptake in both transgenic and wild-type sections, qualifying the cerebellum as a reference region. To verify the specific interaction between the radiotracer and its target poly-GA, we performed a blocking experiment. The brain sections of transgenic mice were treated with the radiotracer in the presence of a 1000-fold excess of unlabeled antibodies, which resulted in a low-level homogeneous background signal (Figure 2g) similar to that of nontransgenic controls. Thus, [^64^Cu]Cu-NODAGA-mAb1A12 shows high specificity for its target poly-GA in mouse tissues.

To obtain a quantitative readout from the *in vitro* autoradiography experiments, the cortex-to-cerebellum ratio (Ratio_CTX/CBL_) was calculated in brain sections from transgenic (*n* = 6) and wild-type (*n* = 4) mice. The log_10_ (Ratio_CTX/CBL_) was 0.05 ± 0.09 (mean ± SD) for Tg and −0.03 ± 0.05 for WT mice (Figure 2h).

### PET Imaging 20 h Post-Injection Shows the Strongest Difference between Tg and WT Mice

**Figure 3:**
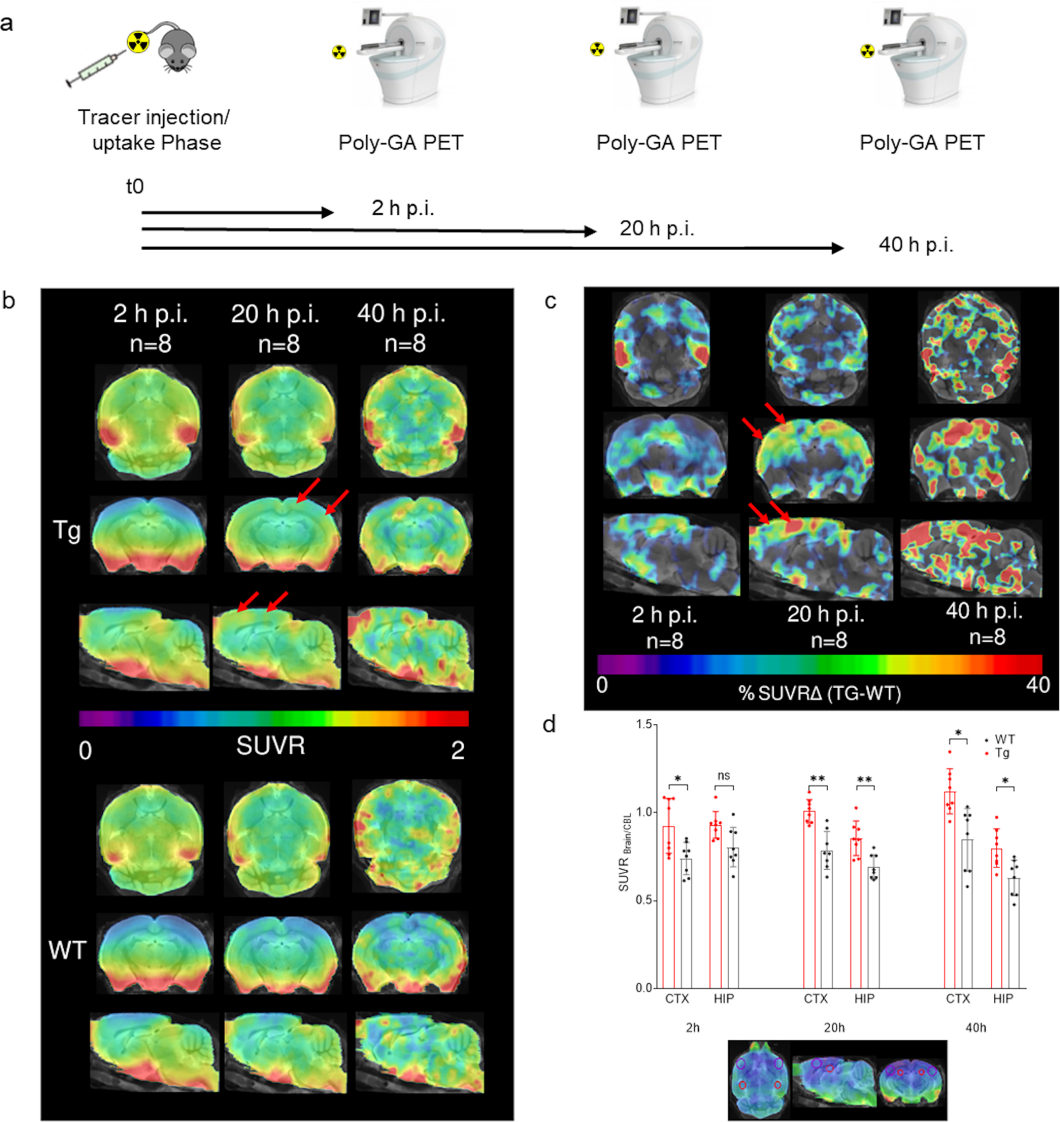
[^64^Cu]Cu-NODAGA-mAb1A12 specifically detects neocortical poly-GA pathology in transgenic mice by PET. (a) Graphical representation of the PET study design. (b) PET SUVR images of the brains of Tg vs WT mice (*n* = 8) at 2, 20, and 40 h p.i. The color code shows the mean PET signal normalized to that of the cerebellum. Note that the basal brain regions initially take up antibodies regardless of genotype. At 20 h p.i., clear enrichment of the PET tracer is seen in the neocortex (arrows). (c) Differential PET images (% SUVRΔ) showing transgene-specific enrichment in the cortical areas (arrows). (d) SUVR_Brain/CBL_ in the cortex and hippocampus of Tg and WT mice, measured by PET at 2, 20, and 40 h p.i. (*n* = 8) (mean ± SD, two-way analysis of variance (ANOVA) and Sidak′s test for multiple comparisons, *p* ≤ 0.05 (*) and ≤0.01 (**)). Circles in the images below the graph indicate the VOIs (volume of interests) used to quantify tracer uptake (purple: CTX; red: HIP).

To assess the novel poly-GA tracer *in vivo*, we used combined PET/CT in poly-GA transgenic and wild-type mice and analyzed the kinetics of CNS uptake in a time series covering 2, 20, and 40 h post-injection (p.i.) since the uptake of antibodies across the blood–brain barrier and into aggregate-bearing neurons may be slow (Figure 3a).

Standardized uptake value ratios (SUVR_Brain/CBL_) were used to visualize cortical tracer uptake in the PET images (Figure 3b) (SUV-scaled PET images are shown in the Supporting Figure S4). PET images showed a higher SUVR_Brain/CBL_ ratio in the transgenic mouse brains than in the control group. This result was most evident in axial slices after 20 h p.i., particularly in the cortical band. To further illustrate this result, Figure 3c shows the difference of SUVR_Brain/CBL_ (%ΔTg-WT) in the brains of transgenic and wild-type mice. The strongest differences in SUVR_Brain/CBL_ can be detected in the cortical band 20 h p.i. At 40 h p.i., PET signals became noisy due to low radioactivity levels in the brain, which may be explained by the decay of copper-64 (12.7 h half-life). Nevertheless, patches of enhanced SUVR_Brain/CBL_ difference in the cortex are clearly visible.

Next, we quantified the SUV from the cortex and hippocampus normalized to the SUV in the cerebellum (SUVR_Brain/CBL_) to compare the wild-type and transgenic mice. The VOIs (volume of interests) were carefully chosen to exclude regions rich in the vasculature, such as the ventricles and meninges. The SUVR_Brain/CBL_ in the cortex and hippocampus of transgenic mice was significantly higher (up to 27%) than that in wild-type animals at 20 and 40 h p.i., indicating the sensitivity of this approach in distinguishing pathological from normal physiological states (Figure 3d). The SUVR_Brain/CBL_ in the hippocampus decreased over time, whereas the SUVR_Brain/CBL_ in the cortical band increased. Analysis of variance revealed a significant effect of genotype and time on tracer uptake in each brain area (Supporting Data).

### *Ex Vivo* Analysis Confirms Enrichment of Anti-GA Tracer in the Brain of poly-GA Mice

**Figure 4:**
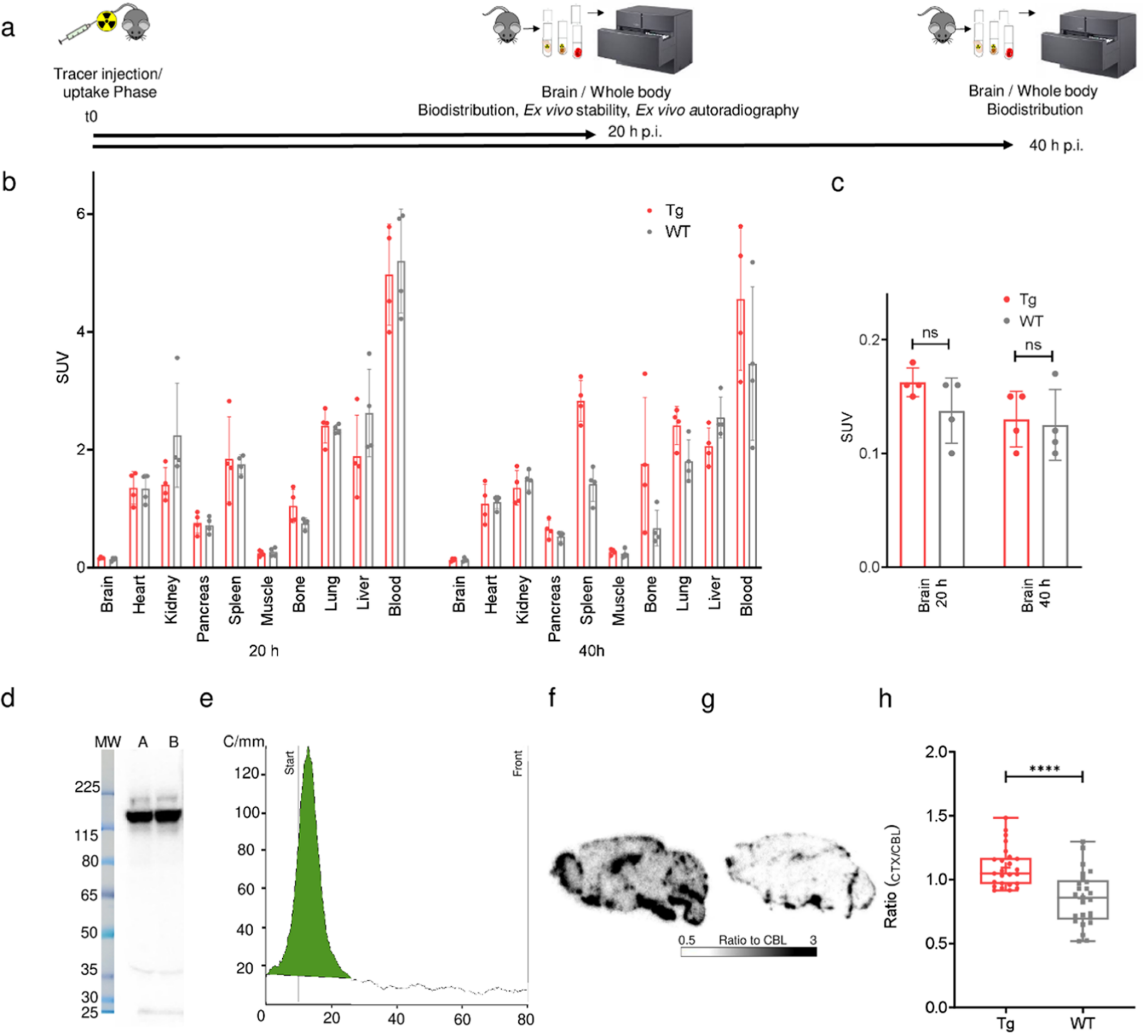
*Ex vivo* evaluation of [^64^Cu]Cu-NODAGA-mAb1A12 shows an intact radiotracer and confirms the regional PET distribution pattern by autoradiography. (a) Graphical representation of the biodistribution study design. (b) SUV of [^64^Cu]Cu-NODAGA-mAb1A12 in organs of WT and Tg mice at 20 and 40 h p.i. (*n* = 4), determined by biodistribution (mean ± SD). (c) SUV of [^64^Cu]Cu-NODAGA-mAb1A12 in the brains of Tg vs WT mice (*n* = 4), measured by biodistribution at 20 and 40 h p.i. (mean ± SD, unpaired *t*-test, *p* > 0.05). (d) Autoradiography of SDS-PAGE gel loaded with plasma from two [^64^Cu]Cu-NODAGA-mAb1A12-injected C57BL/6J mice (A, B). (e) Radio-TLC of plasma from [^64^Cu]Cu-NODAGA-mAb1A12-injected C57BL/6J mice on ITLC-SG chromatography paper, *R*_f_ (tracer) = 0.0–0.1. *Ex vivo* autoradiography of brain sections from (f) transgenic and (g) wild-type mice. (h) Cortex-to-cerebellum ratio from *ex vivo* autoradiography sections of a representative transgenic and wild-type mouse (unpaired *t*-test, *p* ≤ 0.0001).

To further analyze the biodistribution of [^64^Cu]Cu-NODAGA-mAb1A12, the isolated organs of transgenic and wild-type mice were measured in a γ counter at 20 and 40 h p.i., and the tracer uptake was expressed as SUVs (standardized uptake values) (Figure 4a,4b and Supporting Table S2). There was a trend toward higher tracer uptake in the brain of the transgenic mice after 20 h compared to the wild type (Figure 4c). Most of the tracer remained in the blood circulation, as expected for antibodies. Increased tracer uptake was detected in the bone (SUV = 1.76) and spleen (SUV = 2.83) of transgenic mice at 40 h p.i. compared to that at 20 h p.i. (bone: 1.04, spleen: 1.84). Quantification of poly-GA levels in the peripheral organs of Tg and WT mice revealed only low poly-GA concentrations, indicating off-target binding of the antibody in these organs (Supporting Figure S3 and Table S1). To verify that no radiometal was detached from the complex, we performed *ex vivo* stability analysis using SDS-PAGE and radio-TLC of plasma samples from [^64^Cu]Cu-NODAGA-mAb1A12-injected C57BL/6J mice at 20 h p.i. SDS-PAGE revealed an intact radiotracer and hardly any other proteins that might have been radiolabeled via transchelation (Figure 4d). Moreover, radio-TLC confirmed the absence of unbound copper-64 (Figure 4e). Release of the radiometal would also result in the accumulation of radioactivity in the liver due to its association with enzymes such as ceruloplasmin.^28^ However, we did not observe any significant accumulation of radioactivity in the liver over time. Thus, we conclude that the complex is stable *in vivo* within the time of investigation and decomplexation does not occur.

To confirm CNS exposure to the 1A12 antibody, we performed a pharmacokinetic study in wild-type C57BL/6JOlaHsd mice that received a single dose of 1A12 antibody (IgG1, IgG2a, or IgG2c subtype, 30 mg/kg, s.c.). For this study, mice were extensively perfused with PBS prior to tissue collection before measuring antibody levels by immunoassay for up to 28 days after administration (Supporting Figure S5 and Table S3). Pharmacokinetic data unequivocally prove CNS exposure of the IgG2a isotype 1A12 antibody. 1A12 variants of the mIgG1 and mIgG2c isotypes also reached the CNS but at lower levels.

To validate the PET results, we performed *ex vivo* autoradiography on transgenic and wild-type mouse brain sections after 20 h p.i. and the final PET/CT scans (Figure 4f,4g). Visual assessment was impaired by interference signals from the vessels. To account for the uptake of the radiotracer in the brain parenchyma, we drew regions of interest excluding the vessels, and cortex-to-cerebellum ratios were calculated. Quantitative analysis of 25 sections of a representative mouse from each group confirmed a higher cortex-to-cerebellum ratio in transgenic mice compared to the wild-type control (1.09 ± 0.15 vs 0.86 ± 0.21, mean ± SD) (Figure 4h), which is consistent with the results from IHC and *ex vivo* poly-GA quantification in various brain regions by immunoassay.

In this study, we developed and evaluated a ^64^Cu-labeled monoclonal anti-GA antibody (mAb1A12) as a novel tracer for the PET imaging of *C9orf72* FTD/ALS. The tracer clearly showed an increased signal in the brain of poly-GA-expressing mice *in vivo*, consistent with the beneficial effects observed in anti-GA immunotherapy strategies,^15,17,22^ but due to limited penetration of the blood–brain barrier, the signal-to-noise ratio was rather low.

PET tracers for Aβ (such as PiB) have been instrumental as pharmacodynamic markers in large clinical trials to evaluate the efficacy of treatments aimed at reducing brain Aβ-deposits.^29^ While small-molecule tracers for intracellular Tau are approaching clinical use, the development of suitable PET tracers for other less-abundant protein aggregates associated with neurodegenerative diseases (NDD), such as α-synuclein and TDP-43, has proven to be more challenging, which may be explained by the lower amounts compared to Aβ and Tau.

Monoclonal antibodies are one of the most important classes of biological agents used for targeted therapy and diagnostics. They exhibit several advantages, such as high affinity and specificity for their target and low background signal.^30,31^ Because highly specific antibodies can be raised against almost any target molecule, monoclonal antibodies have become increasingly popular for molecular imaging and radionuclide therapy.^30,32^ However, the large size of antibodies (150 kDa) greatly limits delivery across the blood–brain barrier (BBB).^30^ However, 0.1–0.5% of unmodified antibodies reach the CNS, and the Aβ antibodies aducanumab and lecanemab have been approved for the treatment of Alzheimer’s disease. Antibodies targeting intraneuronal aggregates are in clinical trials for Tau and α-synuclein.^7,33^ Attempts have been made to facilitate delivery across the BBB, for example, by targeting the transferrin receptor (TfR). Transferrin receptor ligands promote the active transport of large cargo via transcytosis across brain endothelial cells. As a result, high-resolution PET/CT images can be obtained with radiolabeled bispecific antibodies, e.g., targeting Aβ.^30,34^ A similar strategy would likely improve the signal-to-noise ratio for our tracer.

Two important factors to be considered in the development of a new tracer based on antibodies are the choice of the radiometal and a suitable chelator since the high stability of the radiometal–chelator complex *in vivo* and the half-life of the radionuclide are important factors given the slow and inefficient delivery to the CNS.^35^ Zr-89 is very common to radiolabel antibodies due to its long half-life, and ^89^Zr-DFO* complexes demonstrate good *in vivo* stability and have been applied in numerous clinical studies. However, high-energy γ emissions with high abundance in combination with a longer half-life are rather unfavorable in terms of absorbed doses. In this study, we selected the positron emitter copper-64, due to its half-life of 12.7 h, which enables imaging also at late time points, and its low positron energy, which results in high-resolution images. Moreover, its coordination chemistry is well understood, and several available chelators form complexes that are stable *in vivo*.^28,35^ We conjugated the mAb1A12 antibody to *p*-NCS-benzyl-NODAGA since antibody modification and radiolabeling with copper-64 can be achieved under mild conditions, especially innocuous pH. In addition, the [^64^Cu]Cu-NODAGA complex is highly stable *in vivo*. Radiolabeling was performed with high RCY and high RCP. The tracer was also shown to be stable for at least 24 h in murine plasma. The antibody was modified in a nonselective fashion, with the inherent risk of compromising target binding if the chelator interacted with the antibody epitope. However, ELISA confirmed that the binding affinity was not impaired after chemical modification. *In vitro* autoradiography showed tracer accumulation primarily in the neocortex and hippocampus of transgenic mice, consistent with immunohistochemical staining and the known Camk2a-Cre expression pattern, resulting in high poly-GA levels in these regions. The cerebellum was chosen as an internal reference region since the expression of poly-GA is low in this brain area and is not susceptible to any significant variation between animals. Therefore, the calculation of SUV ratios (SUVR) is a valuable tool for quantifying region-specific tracer uptake in the brain. The SUVR values were significantly higher in Tg mice than in WT mice. Tracer binding to the poly-GA inclusions is highly specific, as shown by a blocking experiment in the presence of an excess of native antibodies.

Based on the favorable *in vitro* characteristics, tracer pharmacokinetics were assessed *in vivo* using a novel transgenic mouse model that allows conditional expression of GFP-(GA)_175_ in excitatory neurons in the neocortex, hippocampus, and striatum using Camk2a-Cre because widespread expression in the CNS using Nestin-Cre resulted in a severe phenotype that would make PET/CT studies difficult.^26^ Using Camk2a-Cre, mice develop cognitive deficits and reach the humane endpoint around 30 weeks of age compared to approximately 6 weeks of age for Nestin-Cre (in-depth characterization in preparation, Zhou et al.). The PET/CT imaging revealed increasing and most prominent enrichment of the [^64^Cu]Cu-NODAGA-mAb1A12 tracer in the cortex of transgenic mice over time compared to that of WT, represented by higher SUVR_Brain/CBL_ values. Both genotype and time significantly influence tracer uptake. PET images also indicate that 20 h p.i. is a favorable time point for image acquisition. Differential SUVR PET/CT images at 20 h p.i. show nearly optimal tracer distribution, whereas very early time points fail to identify clear distribution differences due to the slow uptake of antibodies across the BBB. Late time points are less useful due to the decay of ^64^Cu, resulting in low radioactivity levels and low signal-to-noise ratios, leading to inferior image quality and poor accuracy of PET data. Relative differences in the images between transgenic and control mice are comparable to those seen with established amyloid-β radiotracers used for PET imaging in Alzheimer′s disease.^36,37^ Moreover, our data were obtained using a small-animal PET scanner with limited spatial resolution compared to human imaging. Due to the low resolution of PET/CT, we cannot distinguish between intracellular uptake of antibodies and binding of antibodies to extracellular poly-GA transmitted between cells.^17^ These data support the development of an active and passive immunization therapeutic approach for poly-GA by showing target engagement in vivo.^15,22^

Consistent with the known poor penetration of antibodies through the BBB, tracer uptake in the brain is rather low. Still, our single-dose pharmacokinetic study proved that the antibody is able to penetrate the BBB, supporting the observation that the radiotracer enters the brain in sufficient amounts to obtain robust PET signals, despite high antibody levels in the vasculature. Whereas biodistribution revealed only a trend toward higher CNS retention in Tg animals 20 h p.i., PET and *ex vivo* autoradiography showed a significant difference between the two groups. The lack of significant biodistribution differences between genotypes at the group level is reasonable since regional analysis by PET and autoradiography allows for a local assessment of tracer uptake alterations, whereas biodistribution accounts for the overall tracer uptake in the brain, including basal activity levels. This shows that PET is an extremely valuable tool to detect regional differences in brain uptake, which cannot be achieved using other methods.

Biodistribution showed higher signals in the bone and spleen of transgenic mice after 40 h compared to 20 h post-injection. Presumably, the tracer also accumulates in the hematopoietic system over time, which may be driven by off-target binding of the radiotracer since poly-GA levels in the peripheral tissue were negligible. The antibody levels in the whole blood of Tg mice were also high. However, *ex vivo* autoradiography demonstrated a significant difference in the cortex/cerebellum ratio, clearly demonstrating that the uptake can be attributed to specific binding to poly-GA aggregates and that it is not just perfusion-driven. IHC and biochemical assessment of poly-GA levels confirm high target expression in brain regions, consistent with the expression pattern of the Camk2a-Cre driver.^27^ These data fully corroborate the increased signals in PET and *ex vivo* autoradiography, thus providing strong evidence that the antibody enters the brain and that the signal is not derived from the vasculature.

The distribution of poly-GA in our mouse model is different from human *C9orf72* ALS/FTD because the cerebellum is largely spared in these mice.^4,13^ The mouse model may show more peripheral expression than patients, who express poly-GA mainly in the CNS and to a lesser extent in the muscle and testis.^13,38^ In *C9orf72* patients, poly-GA pathology is highly abundant in the neocortex, hippocampus, and thalamus.^21^ It would be interesting to test whether the onset of poly-GA pathology in *C9orf72* mutation carriers correlates with the prodromal atrophy reported in these regions.^3^ PET may also be useful to track poly-GA pathology in therapeutic studies in future clinical trials. Therefore, clinical translation of a poly-GA tracer is highly desired; however, improving BBB transmission through a shuttle system will likely be required to improve the signal-to-noise ratio for clinical use.

## Conclusions

Herein, we present the first poly-GA targeting radiotracer and provide comprehensive *in vitro*, *in vivo*, and *ex vivo* data demonstrating that [^64^Cu]Cu-NODAGA-mAb1A12 is a useful tracer for preclinical *C9orf72* ALS/FTD imaging. Despite low BBB penetration, high-quality images were obtained. To enhance tracer uptake in the brain, the 1A12 antibody can be engineered for receptor-mediated transcytosis via the TfR.

## Materials and Methods

The supporting information provides detailed information on the materials and experimental procedures, including methods, figures, and data for ELISA, SDS-PAGE, *in vitro* stability experiments, immunohistochemistry, PET/CT statistics, the arsenazo spectrometric assay, poly-GA immunoassay, the pharmacokinetics study, biodistribution, HPLC, radio-TLC chromatograms, and SUV-scaled PET images.

### Animals

Floxed GA-stop mice were described previously and maintained at the C57BL/6J background. Briefly, GFP-(GA)_175_ genes encoding nonrepeating alternate codons downstream of a floxed stop cassette encoding a puromycin resistance gene followed by SV40 polyadenylation signal were integrated at the Rosa26 safe harbor.^26^ Crossing GA-stop with Camk2a-Cre (Tg(Camk2a-cre)93Kln)^27^ and subsequent excision of the stop cassette resulted in DPR expression throughout Camk2a-positive excitatory neurons (ROSA26 GFP-(GA)_175_ ±; Camk2a-Cre ±). The full phenotypic characterization of this mouse line will be reported in another article, which is currently under preparation. We compared (ROSA26 GFP-(GA)_175_ ±; Camk2a-Cre ±) transgenic mice (Tg) and GA± Camk2a-Cre −/– littermate controls that did not express poly-GA referred to as wild-type (WT) mice (4–7 months, 15–33 g).

### Antibody Modification

The mAb1A12 antibody modification was performed with *p*-NCS-benzyl-NODAGA (2,2′-(7-(1-carboxy-4-((4-isothiocyanatobenzyl)amino)-4-oxobutyl)-1,4,7-triazonane-1,4-diyl)diacetic acid, CheMatech, Macrocycle Design Technologies) in metal-free phosphate buffer (pH 8.5, 0.1 M) as follows. The 1A12 antibody (4 mg) was dissolved in 1 mL of metal-free phosphate buffer (0.1 M, pH 8.5). An excess amount of dissolved *p*-NCS-benzyl-NODAGA (3.8 mg, 100 μL, 7.3 μmol) in metal-free phosphate buffer (0.1 M, pH 8.5) was added to the antibody solution. The reaction mixture was incubated overnight at 4 °C.^39^ The mixture was purified using either Microcon centrifugal filter units (Ultracel 30 kDa, 0.5 mL, Merck Millipore Ltd.) or Amicon ultracentrifugal filter units (Ultracel 30 kDa, 0.5 mL, Merck Millipore Ltd.). The buffer was changed to metal-free ammonium acetate (0.5 M, pH 6.8) by centrifugation (20,817*g*, 20 min, 4 °C, Centrifuge 5810 R, Eppendorf). Quality control of the conjugated antibody was performed by HPLC (Agilent Technologies, 1200 series) using a Phenomenex column (BioSep 5 μm SEC-s 4000 500 Å LC Column 300 mm × 7.8 mm) with 0.1 M sodium phosphate buffer (pH 7.2, isocratic run, 1 mL/min, 20–30 min). NODAGA-mAb1A12 eluted at *R*_t_ = 10.0 min, as detected by UV adsorption (280 nm).

### Radiolabeling

NODAGA-mAb1A12 was centrifuged (5 min, 20,817*g*, 4 °C, Ultracel 30 kDa, 0.5 mL, Merck Millipore Ltd.) in order to decrease the reaction volume to 200 μL of ammonium acetate buffer (0.5 M, pH 6.8) before labeling. Next, 100–150 MBq [^64^Cu]CuCl_2_ was added to NODAGA-mAb1A12 (100 μg). The pH was adjusted to 5.6 using ammonium acetate buffer (0.1 M, pH 5.5). The mixture was incubated for 30–45 min, with gentle shaking at 400 r.p.m. at 42 °C. The reaction mixture was purified using Microcon Centrifugal filter units, and the buffer was changed to phosphate-buffered saline (ABX) for injection into mice. For quality control, radio-TLC on ITLC-SG glass microfiber chromatography paper (Agilent Technologies, Folsom, CA) was performed with sodium citrate buffer (0.1 M, pH 5.0) as the mobile phase (*R*_f_ (tracer) = 0.0, *R*_f_ ([^64^Cu]CuCl_2_) = 0.9, *R*_f_ ([^64^Cu]Cu-NODAGA) = 0.5). Furthermore, radio-HPLC showed a retention time of 11.0 min for [^64^Cu]Cu-NODAGA-mAb1A12.

### Autoradiography

Brain sections from GA ± Camk2a ± Tg vs WT mice were deparaffinized in xylene followed by washing with 100, 99, 95, and 70% ethanol and water. The sections were preincubated in 50 mM Tris-HCl (pH 7.4, RT, 10 min) and then incubated with a maximum of 1 MBq/mL of [^64^Cu]Cu-NODAGA-mAb1A12 in phosphate buffer (pH 7, 60 min, RT). To determine specificity, brain sections from GA ± Camk2a ± Tg mice were preincubated with a 1000-fold excess of nonlabeled mAb1A12 (300 μg) in the presence of ∼0.3 MBq [^64^Cu]Cu-NODAGA-mAb1A12 (0.3 μg). The sections were washed with cold Tris-HCl + 5% ethanol (pH 7.4, 1 × 5 min, 4 °C), followed by distilled water (RT, 5 s). Finally, the sections were dried at RT for 1 h. *Ex vivo* autoradiography was performed on the transgenic and wild-type mice after 20 h p.i. After the PET/CT scan, one hemisphere of the brain was isolated and fixed on a polymer (Tissue-Tek O.C.T. compound). The brain was frozen at −20 °C and cut into sagittal sections of 20 μm thickness using a Leica CM 1860 cryostat machine. All brain sections were exposed to a phosphor imaging plate for 24 to 30 h in the dark at RT and then scanned with a CR-Reader (CR35 BIO, DÜRR MEDICAL). The images were analyzed using Aida Image Analyzer software (v.4.50.010, Elysia-raytest GmbH). A manually drawn region of interest (ROI) was placed in the cerebellum as a pseudoreferencing tissue. After background subtraction, intensity normalization of all sections was performed by calculating the brain-to-cerebellum (CBL) ratios.

### Small-Animal PET/CT Imaging

Mice (4–7 months) were injected with 9.7 ± 2.4 MBq of [^64^Cu]Cu-NODAGA-mAb1A12 (corresponding to 10.3 ± 3.0 μg per mouse) in 200 μL of phosphate buffer via the lateral tail vein under isoflurane anesthesia. The mice (*n* = 8) were scanned first with CT (70kVp/650 μA, exposure time 300 ms, Helical 1.0 pitch). The scan was followed by 30 min static animal PET imaging (with coincidence mode 1–5 in 1 scan position) 2, 20, and 40 h post-injection (p.i.). Small-animal PET imaging was carried out under constant anesthesia with isoflurane (1.5% at 1.5 L flow per minute) with a Mediso Nanoscan PET/CT (Budapest, Hungary). PET/CT images were reconstructed using the Tera Tomo 3D algorithm (4 iterations and 6 subsets) and analyzed using PMOD (version 3.5; PMOD Technologies Ltd., Zurich, Switzerland). PET images of each mouse were co-registered with CT images of the corresponding mice. The images were aligned to the magnetic resonance imaging (MRI) mouse brain atlas. Volumes of interest (VOIs) were drawn to assess tracer enrichment in different brain regions (from the atlas and manually as spheres). Standard uptake value (SUV) ratios were calculated by dividing the SUV of interest by that of the cerebellum. Average and differential (%SUVRΔ (Tg-WT)) PET images were generated from all measured mice (*n* = 8 per group) at 2 h, 20 and 40 h p.i.

### Biodistribution

The biodistribution of [^64^Cu]Cu-NODAGA-mAb1A12 was studied in GA ± Camk2a ± Tg vs WT mice at 20 and 40 h p.i. (*n* = 4). After PET/CT scanning, the mice were transferred to the anesthesia box for isoflurane overdose and euthanized by cervical dislocation. Mouse organs including the brain, heart, kidneys, liver, bone, muscle, spleen, pancreas, lungs, and blood were collected for biodistribution analysis. The brain was divided into two hemispheres. One hemisphere was used for biodistribution together with the other organs. The mass and radioactivity of each organ were measured in a γ counter (HIDEX AMG γ Counter, version 1.6.0.0, counting time of 1 min, counting window of 450–570 keV).

### Statistical Analysis

Statistical analyses were conducted using GraphPad Prism 8.4.3 and 9. Unpaired Student′s *t*-test, two-way ANOVA, or pairwise Sidak’s multiple comparison tests were performed, as indicated in the captions. Statistical significance was set at *p* > 0.05 (ns), ≤ 0.05 (*), ≤ 0.01 (**), ≤ 0.001 (***), and ≤0.0001 (****).

## Supporting Information Available

Materials, supporting methods for ELISA, SDS-PAGE, *in vitro* stability experiments, immunohistochemistry, and PET/CT statistics; figures of the arsenazo spectrometric assay, HPLC and radio-TLC chromatograms, poly-GA immunoassay, SUV-scaled PET images, and pharmacokinetic studies; data on poly-GA concentrations in tissue, biodistribution, and pharmacokinetic studies (PDF)

## Notes

Animal handling and experiments in this study were performed under the supervision of a veterinarian in accordance with the German Animal Welfare Law and were approved by the Government of Upper Bavaria, Germany (vet_2–17–106).

## Abbreviations

FTD: frontotemporal dementia
ALS: amyotrophic lateral sclerosis
PET/CT: positron emission tomography/computed tomography
SUVR: standardized uptake value ratio
VOI: volume of interest
RCP: radiochemical purity
RCY: radiochemical yield
HPLC: high-performance liquid chromatography
TLC: thin-layer chromatography
IHC: immunohistochemistry
CNS: central nervous system
TfR: transferrin receptor
BBB: blood–brain barrier
