# Supplementary material for: Development and Preclinical Evaluation of a Copper-64-Labeled Antibody Targeting Glycine-Alanine Dipeptides for PET Imaging of C9orf72-Associated Amyotrophic Lateral Sclerosis/Frontotemporal Dementia
Source: ACS Pharmacol Transl Sci. 2024 Apr 25;7(5):1404–14. doi: 10.1021/acsptsci.4c00037 (PMC11091963; doi:10.1021/acsptsci.4c00037)
Supplement: Supplementary file 1 — pt4c00037_si_001.pdf [file pt4c00037_si_001.pdf]

Development and preclinical evaluation of a copper-64 labeled antibody targeting glycine-alanine dipeptides for PET imaging of *C9orf72* associated amyotrophic lateral sclerosis / frontotemporal dementia

Monireh Shojaei[1], Qihui Zhou[2], Giovanna Palumbo[1], Rebecca Schaefer[1], Janne Kaskinoro[3], Pirjo Vehmaan-Kreula[3], Peter Bartenstein[1,4], Matthias Brendel[1,2,4], Dieter Edbauer[2,4], Simon Lindner[1]*

1 Department of Nuclear Medicine, University Hospital, LMU Munich, 81377 Munich, Germany

2 German Center for Neurodegenerative Diseases (DZNE), 81377 Munich, Germany

3 Orion Corporation Orion Pharma, 02200 Espoo, Finland

4 Munich Cluster for Systems Neurology (SyNergy), 81377 Munich, Germany

*Corresponding author: simon.lindner@med.uni-muenchen.de

Supplemental Data

SUPPLEMENTAL METHODS

MATERIALS

Chemicals and materials were purchased from Merck, VWR, Thermo Fisher Scientific, CheMatech, Roche, Agilent, Eppendorf, and Sigma Aldrich, and were used as received without any purification. [^{64}Cu]$CuCl_2$ was obtained from the Department of Preclinical Imaging and Radiopharmacy, University Hospital Tuebingen, Germany.

ELISA

The Nunc MaxiSorp™ flat-bottom 96-well plates were incubated with recombinant GST-$(GA)_{15}$ produced in E. coli [1, 2] in PBS overnight at 4°C at various concentrations (0.5. 0.1, 0.02, 0.004, 0.0008, 0.00016, 0.00032 and 0 ng/µl). After 1 h incubation in blocking solution (1% BSA, 0.05% Tween 20 in PBS), 0.5 µg/ml of mAb1A12 or NODAGA-mAb1A12 were added for 1 h. After three washes with 0.05% Tween 20 in PBS, anti-GA antibodies were detected with anti-mouse HRP. 3,3′,5,5′-Tetramethylbenzidine was used as chromogenic substrate. After stopping the reaction with 2 M H_2SO_4, the absorbance was read at 450 nm. Two technical replicates were measured for each sample. EC_{50} values were calculated using Quest Graph EC_{50} Calculator, AAT Bioquest, Inc.

SDS-PAGE

Antibody integrity was assessed by SDS-PAGE under non-reducing conditions. Unmodified antibody mAb1A12, modified antibody NODAGA-mAb1A12 and labeled antibody [^{64}Cu]Cu-NODAGA-mAb1A12 (0.4 µg, PBS) were incubated with SDS sample buffer at 90°C for 10 min, loaded onto an 8% Bolt Bis-Tris Plus gel and run with MOPS buffer at 200V for 35 min. Spectra™ Multicolor Broad Range Protein Ladder (Thermo Fisher Scientific) served as standard. Coomassie staining (SimplyBlue Safestain, Thermo Fisher Scientific) was performed overnight. 2 mL of 20% aq. NaCl (w/v) were added to 20 mL of stain to avoid decrease of sensitivity. Radioactive read out of the gel was done via radio-TLC (miniGita, raytest, iTLC-SG, 0.1 M sodium citrate buffer pH 5) and autoradiography. The gel was exposed to a phosphorimaging plate for four hours, scanned with a CR-Reader (CR35 BIO, Dürr Medical), and analyzed using Aida Image Analyzer software.

Antibody integrity was analysed *ex vivo* in plasma samples from [^{64}Cu]Cu-NODAGA-mAb1A12 (46.0 ± 4.1 MBq, C57BL/6J, n = 3, 2 males and 1 female) injected mice 20h p.i. Blood was collected from the heart in EDTA tubes (Sarstedt Microvette 100 K3E). Tubes were centrifuged (3000 × g, 5 min) and the top layer (plasma) separated. Plasma concentrations were quantified by radioactivity measurements using a gamma counter. SDS-PAGE was performed as previously described, with the exception that 0.13 µg of antibody was loaded per well. Autoradiography and radio-TLC were performed accordingly.

IN VITRO STABILITY

The stability of ^{64}Cu-labeled NODAGA-mAb1A12 was determined in mouse plasma. Anaesthetized mice (isoflurane) were killed by cervical dislocation. Then, murine blood was collected by cardiac puncture and transferred to an Eppendorf tube. Murine plasma was separated from blood cells by centrifugation (Mini spin centrifuge, Eppendorf) for 10 min at 3000 × g and then stored at -20 °C. 113 MBq of [^{64}Cu]Cu-NODAGA-mAb1A12 in 70 µL phosphate-buffered saline were incubated in 200 µL murine plasma

(gentle shaking at 400 r.p.m, 37°C, 24 h). 10 µL of sample were taken at 30 min, 1 h, 1.5 h, 2 h, 15 h, 20 h, and 24 h for stability measurements using HPLC (Agilent Technologies, 1200 series, Phenomenex column, BioSep ™ 5 µm SEC-s 4000 500 Å LC Column 300 x 7.8 mm, with 0.1 M sodium phosphate buffer, pH 7.2, isocratic run, 1 ml/min, 20 - 30 min).

IMMUNOHISTOCHEMISTRY

Immunohistochemistry was performed on 5 µm paraffin-embedded tissue sections as described previously [3] using the following antibodies: GFP (632381, Takara 1: 250), poly-GA (1A12, IgG1 mouse, purified and biotinylated, 1:500), 1x Anti-HA high Affinity Rat monoclonal antibody clone 3F10 (11867423001, Roche), recombinant anti-mouse IgG2a antibody [RM107] (ab190463, Abcam). After deparaffinization and dehydration, heat-induced antigen retrieval was performed with citrate buffer (pH 6) for 20 min in the steamer. Slides were then blocked and incubated with primary antibody overnight at 4°C. Slides were washed and detected with the DCS supervision 2 Kit (DCS innovative diagnostic-system) according to the manufacturer's instructions. Bright-field images were taken on a Leica DMi8 fluorescence microscope (Leica).

STATISTICAL ANALYSIS OF SUVRs FROM PET/CT IMAGES

The results (Two-way ANOVA) revealed that genotype alone has a significant effect in CTX ($F(1,14) = 22.92$, $p = 0.0003$) and HIP ($F(1,14) = 20.34$, $p = 0.0005$) on tracer uptake. Additionally, time shows a significant correlation with tracer uptake in CTX ($F(1.717, 24.04) = 8.710$, $p = 0.0021$) and HIP ($F(1.835, 25.69) = 14.11$, $p = 0.0001$. A significant interaction effect between genotype and time in the CTX ($F(2,28) = 0.7942$, $p = 0.4618$) and HIP (($F(2,28) = 0.3210$, $p = 0.7281$) could not be observed. Pairwise Sidak's multiple comparison tests were conducted to examine differences between the genotype at each time point for each brain region (Fig. 3d). We found that there were statistically significant differences in tracer uptake between the genotype in CTX at 2 h p.i. ($p = 0.0416$), at 20 h p.i. ($p = 0.0011$) and at 40 h p.i. ($p = 0.0106$), and in HIP at 20 h p.i. ($p = 0.0065$) and at 40 h p.i. ($p = 0.0181$). No significant differences were observed at 2 h p.i. ($p = 0.0637$) in HIP.

SUPPLEMENTAL FIGURES

FIG. S1 ARSENAZO SPECTROMETRIC ASSAY

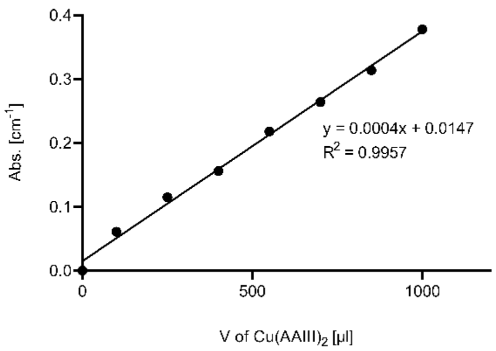

Validation of Lambert Beer's law. The adsorption was measured at different concentrations of $Cu(AAIII)_2$ in a solution of 0.15 M NH_4OAc at pH 7.0 in a 1.0 mL cuvette using a UV-Vis spectrophotometer. Linear regression, $R^2 = 0.9957$.

FIG. S2 HPLC AND RADIO-TLC

A

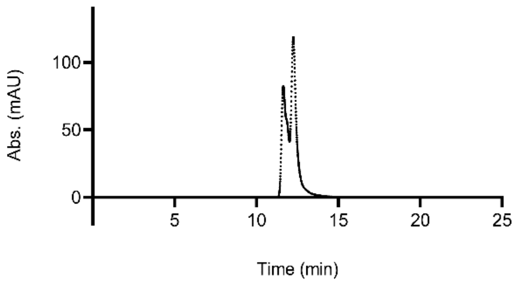

B

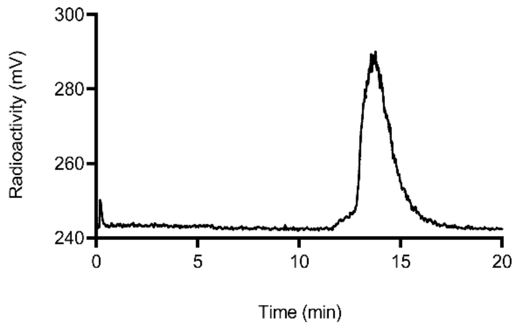

C

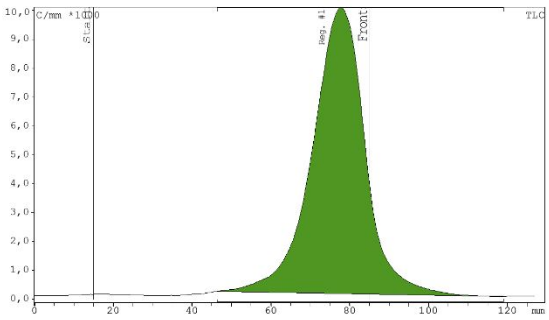

D

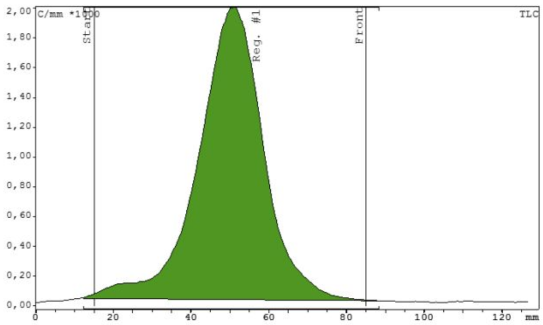

(A) HPLC chromatogram of *p*-NCS-benzyl-NODAGA, $R_t = 13.6$ to 14.2 min at 280 nm (UV channel). (B) HPLC chromatogram of [^{64}Cu]Cu-NODAGA, $R_t = 13.8$ min (radio channel). (C) Radio-TLC of [^{64}Cu]$CuCl_2$ on ITLC-SG chromatography paper, $R_f = 0.9$. (D) Radio-TLC of [^{64}Cu]Cu-NODAGA on ITLC-SG chromatography paper, $R_f = 0.5$.

FIG. S3 POLY-GA IMMUNOASSAY

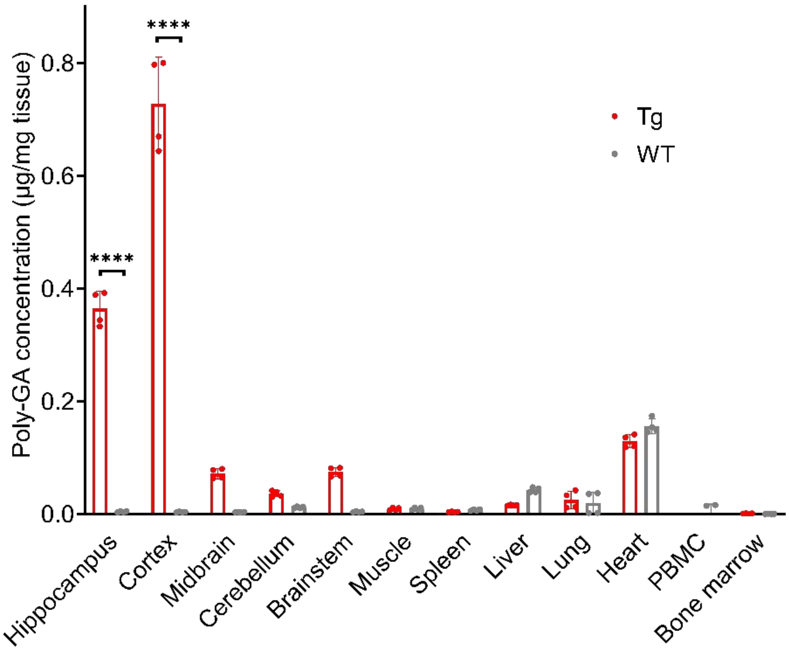

The poly-GA immunoassay was performed to measure poly-GA in different tissue of GA-CFP mice as described previously with slight modification (Zhou et al, 2020). Mouse tissue samples were homogenized in 400µl to 600µl of RIPA buffer (137 mM NaCl, 20 mM Tris pH 7.5, 10% glycine, 1% Triton X-100, 0.5% Na-deoxycholate, 0.1% SDS, 2 mM EDTA, protease and phosphatase inhibitors, benzonase nuclease) using homogenizer (Precellys). Samples were incubated with shaking at 4°C for 20 min and centrifuged at 13,000 g for 10 min at 4°C. Supernatant was then collected for immunoassay analysis and protein concentration was determined by Bradford assay. Streptavidin Gold multi-array 96-well plates were incubated with biotinylated anti-GA clone 1A12 overnight at 4°C and blocked with 1% BSA, 0.05% Tween 20 in PBS for 1 h. Equal amounts of samples were added in duplicate wells for 1.5 h, followed by 1.5 h incubation with the secondary sulfo-tag labeled α-GA clone 1A12. Serial dilution of recombinant RR-$(GA)_8$-RR in blocking buffer was used to generate a standard curve. The intensity of emitted light upon electrochemical stimulation was measured using the MSD QuickPlex 520, and the background was corrected by the average response obtained from blank wells. Data are given as mean ± SD, unpaired t-test, $p \leq 0.0001$.

FIG. S4 SUV SCALED PET IMAGES

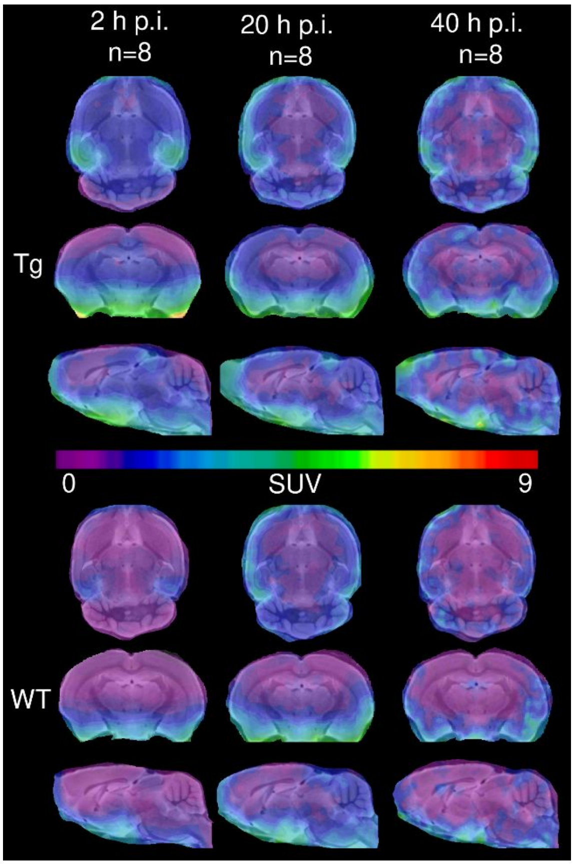

Tracer uptake in brains of Tg and WT mice at 2 h p.i. (left), 20 h p.i. (center) and 40 h p.i. (right) expressed as standard uptake values (SUV).

FIG. S5 CNS EXPOSURE OF THE 1A12 ANTIBODY

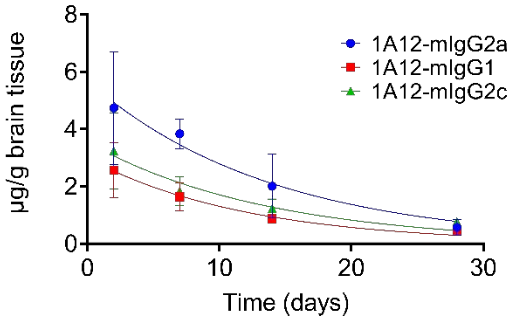

Study Design

The pharmacokinetics of 1A12 antibodies was investigated in plasma and brain of C57BL/6JOlaHsd mice (male, 3-6 animals/group). Mice were administered a single dose (30 mg/kg s.c.) of 1A12 antibodies of different IgG isotypes (IgG1, IgG2a, IgG2c). Sampling times for plasma were 0.5 h, 6 h, 1 d, 2 d, 3 d, 7 d, 14 d, 21 d, 28 d p.i. and for brain tissue 2 d, 7 d, 14 d and 28 d p.i. (compare Table S3). Before tissue collection mice were perfused with PBS. Samples were analyzed by anti-DPR ELISA using GST-$(GA)_{15}$ antigen.

Anti-DPR ELISA

Plasma samples were diluted with 0.1% Tween in PBS (1:50000 – 1:1000000), brain was homogenized into 10 × v/w, and concentration was adjusted to 10-14 mg/mL (1:10-1:40 dilution with 0.1% Tween in PBS). 96-well covalent binding plates (Thermo Fisher Scientific) were coated overnight at 4°C with 10 ng GST-$(GA)_{15}$ per well. After 1 hour blocking (PBS with 2% BSA and 0.1% Tween-20), serial dilutions of plasma or brain tissue samples of 1A12 antibodies (mIgG1, mIgG2a, mIgG2c, 4-9000 pg/mL) were added to the wells, incubated for 2 hours on a shaker, and detected after a 1-hour incubation on a shaker with HRP-conjugated antibodies (rabbit anti-mouse IgG1-HRP, dilution 1:10000; goat anti-mouse IgG2a-HRP, 1:1000; goat anti-mouse IgG2c-HRP, 1:10000). Signals were developed using Pierce™ TMB Substrate Kit (Thermo Fisher Scientific) and detected via spectrophotometry at 450 nm.

SUPPLEMENTAL TABLES

TABLE S1 POLY-GA IMMUNOASSAY DATA

**Poly-GA concentration µg/mg tissue**	**Tg**	**WT**
Hippocampus	0.365 ± 0.026	0.004 ± 0.001
Cortex	0.728 ± 0.072	0.004 ± 0.001
Midbrain	0.072 ± 0.008	0.003 ± 0.000
Cerebellum	0.036 ± 0.005	0.012 ± 0.001
Brainstem	0.075 ± 0.007	0.004 ± 0.001
Muscle	0.009 ± 0.002	0.009 ± 0.002
Spleen	0.004 ± 0.001	0.007 ± 0.001
Liver	0.016 ± 0.001	0.043 ± 0.004
Lung	0.025 ± 0.013	0.019 ± 0.017
Heart	0.129 ± 0.010	0.156 ± 0.011
PBMC	-0.006 ± 0.003	0.001 ± 0.015
Bone marrow	0.001 ± 0.000	0.000 ± 0.000

TABLE S2 BIODISTRIBUTION DATA 20 h AND 40 h p.i.

**SUV Mean ± SD**	**20 h**		**40 h**	
	**Tg**	**WT**	**Tg**	**WT**
Brain	0.16 ± 0.01	0.14 ± 0.03	0.13 ± 0.02	0.13 ± 0.02
Heart	1.35 ± 0.24	1.34 ± 0.21	1.08 ± 0.28	1.11 ± 0.10
Kidney	1.41 ± 0.25	2.24 ± 0.76	1.35 ± 0.25	1.48 ± 0.14
Pancreas	0.76 ± 0.15	0.72 ± 0.12	0.65 ± 0.13	0.53 ± 0.07
Spleen	1.84 ± 0.63	1.75 ± 0.14	2.83 ± 0.30	1.41 ± 0.25
Muscle	0.24 ± 0.04	0.26 ± 0.06	0.27 ± 0.04	0.23 ± 0.06
Bone	1.04 ± 0.25	0.75 ± 0.07	1.76 ± 0.98	0.67 ± 0.26
Lung	2.40 ± 0.25	2.35 ± 0.06	2.41 ± 0.28	1.81 ± 0.32
Liver	1.88 ± 0.61	2.62 ± 0.64	2.06 ± 0.28	2.55 ± 0.30
Blood	4.97 ± 0.74	5.20 ± 0.76	4.55 ± 1.05	3.46 ± 1.13

TABLE S3 PHARMAKOKINETIC DATA OF THE 1A12 ANTIBODY

**Drug**	**Tissue**	**C_{max} (µg/ml, µg/g)**	**C_{last} (µg/ml, µg/g)**	**$AUC_{48\text{-}672}$ (h×µg/ml, h×µg/g)**	**$t_{1/2z}$ (h)**	**$K_{p,\ brain}$ ($AUC_{48\text{-}672}$)**
1A12-mIgG1	Brain	2.57 ± 0.97	0.43 ± 0.04	742	272	0,024
	Plasma	121 ± 38	13 ± 2	31107	202	
1A12-mIgG2a	Brain	4.73 ± 1.97	0.57 ± 0.29	1549	183	0,034
	Plasma	159 ± 28	7 ± 12	45632	121	
1A12-mIgG2c	Brain	3.23 ± 1.33	0.77 ± 0.06	975	411	0,016
	Plasma	215 ± 27	29 ± 1	62522	239	

REFERENCES

(1) Mori, K., Arzberger, T., Grässer, F. A., Gijselinck, I., May, S., Rentzsch, K., Weng, S.-M., Schludi, M. H., van der Zee, J., Cruts, M., Van Broeckhoven, C., Kremmer, E., Kretzschmar, H. A., Haass, C.,Edbauer, D. (**2013**) Bidirectional transcripts of the expanded C9orf72 hexanucleotide repeat are translated into aggregating dipeptide repeat proteins, *Acta Neuropathol. 126*, 881-893. doi:https://doi.org/10.1007/s00401-013-1189-3.

(2) Mori, K., Weng, S.-M., Arzberger, T., May, S., Rentzsch, K., Kremmer, E., Schmid, B., Kretzschmar, H. A., Cruts, M., Van Broeckhoven, C., Haass, C.,Edbauer, D. (**2013**) The C9orf72 GGGGCC Repeat Is Translated into Aggregating Dipeptide-Repeat Proteins in FTLD/ALS, *AAAS Sel. Symp. 339*, 1335-1338. doi:https://doi.org/10.1126/science.1232927

(3) Zhou, Q., Mareljic, N., Michaelsen, M., Parhizkar, S., Heindl, S., Nuscher, B., Farny, D., Czuppa, M., Schludi, C., Graf, A., Krebs, S., Blum, H., Feederle, R., Roth, S., Haass, C., Arzberger, T., Liesz, A.,Edbauer, D. (**2020**) Active poly-GA vaccination prevents microglia activation and motor deficits in a C9orf72 mouse model, *EMBO Mol Med 12*, e10919. doi:https://doi.org/10.15252/emmm.201910919.
